# Supplementary material for: Patterns of Intron Gain and Loss in Fungi
Source: PLoS Biol. 2004 Nov 30;2(12):e422. doi: 10.1371/journal.pbio.0020422 (PMC532390; doi:10.1371/journal.pbio.0020422)
Supplement: Table S1 — Also available at http://genes.mit.edu/NielsenEtAl/. (4.3 MB ZIP). [file pbio.0020422.st001.zip › NielsenEtAl/html/889.html]

AN7603.1.NCU09747.1.MG06108.1.FG05300.1


```
 CLUSTAL W (1.82) Multiple Sequence Alignments - Introns Inserted


Sequence 1: MG06108.1	201 aa
Sequence 2: FG05300.1	201 aa
Sequence 3: NCU09747.1	200 aa
Sequence 4: AN7603.1	200 aa
Alignment Length: 201 aa
Number Identitical Residues: 135 aa
Alignment Score (without introns) 5917


MG06108.1 	MSLTYGLGGTGSALAVVGAY1MLFTGSGESFD0IGATIEEISPFAWASLGIALCIGLSVV
NCU09747.1	MSLTYGAGGIGTSLAVIGAY1MLFTGSGEAFN0VGAFLESVSPYAWADLGIALCIGLSVV
FG05300.1 	MSLYYGVGGIGSFLTIVGGY1MLFTGSGESFD~VGAFLEAVSPYTWATLGIAFCIGLSVV
AN7603.1  	MGLSYPFGFATVSLLVIGGY1MLFHGEGEQFN0VGQFLESVSPYVWANIGIAMCIGLSVV
          	*.* *  *     * ::*.* *** *.** *: :*  :* :**:.** :***:*******

MG06108.1 	GAAW2GIFITGSSILGGGVKAPRIRTKNLISIIFCEVVAIYGVIMAIVFQAKINTLDRTG
NCU09747.1	GAAW2GIFITGSSILGGGVKAPRIRTKNLISIIFCEVVAIYGVIMAIVFSAKLQPVTGAQ
FG05300.1 	GAAW2GIFITGSSILGGGVKAPRIRTKNLISIIFCEVVAIYGVIMAIVFSQKVENVSGAD
AN7603.1  	GAAW2GIFLTGSSIVGGGVKAPRIRTKNLISIIFCEVVAIYGVIMAIVFSSKLNLATGDE
          	**** ***:*****:**********************************. *::      

MG06108.1 	YYSANAYFTGYALFWSGLTVGACNLICGVAVGINGSGAALADAADPTL2FVKILVIEIFS
NCU09747.1	YYSGDSYYTGFALFWAGLTVGMCNLVCGVAVGINGSGAALADAADPTL2FVKILVIEIFS
FG05300.1 	LYAAESYYTGYALFWSGITVGMCNLICGVAVGINGSGAALADAADPSL2FVKILVIEIFS
AN7603.1  	IHSPSSYYTGYALFWGGITVGACNLICGISVGINGSGAALADAADPSL2FVKILVVEIFS
          	 :: .:*:**:****.*:*** ***:**::****************:* ******:****

MG06108.1 	SILGLFGLIIGLLTSGKANDFGSSG
NCU09747.1	SVLGLFGLIVGLLVSGKAPDFGAQ-
FG05300.1 	SVLGLFGLIIGLLVSSKAPAFGEKK
AN7603.1  	SVLGLFGLIVGLLVQGKATEFGAA-
          	*:*******:***...**  **
```
